# ^18^F‐Fluorodeoxyglucose positron emission tomography can be used to determine the indication for endoscopic resection of superficial esophageal cancer

**DOI:** 10.1002/cam4.1628

**Published:** 2018-06-28

**Authors:** Masanobu Nakajima, Hiroto Muroi, Haruka Yokoyama, Maiko Kikuchi, Satoru Yamaguchi, Kinro Sasaki, Hiroyuki Kato

**Affiliations:** ^1^ First Department of Surgery Dokkyo Medical University Mibu Tochigi Japan

**Keywords:** endoscopic resection, endoscopic submucosal dissection, esophageal cancer, FDG‐PET, superficial esophageal cancer

## Abstract

^18^F‐Fluorodeoxyglucose positron emission tomography (FDG‐PET) is a useful imaging modality that reflects the tumor activity. However, FDG‐PET is mainly used for advanced cancer, not superficial cancer. In this study, we investigated the relationship between the superficial tumor depth of esophageal cancer and the FDG uptake to determine the indications for endoscopic resection (ER). From 2009 to 2017, 444 patients with esophageal cancer underwent esophagectomy or endoscopic submucosal dissection (ESD), and 195 patients were pathologically diagnosed with superficial cancer. Among them, 146 patients were examined by FDG‐PET before esophagectomy or ESD. In these 146 patients, the relationship between the pathological tumor depth and FDG uptake was analyzed. The mean maximum standardized uptake value in pT1a‐EP/LPM tumors was 1.362 ± 0.890, that in pT1a‐MM/pT1b‐SM1 tumors was 2.453 ± 1.872, and that in pT1b‐SM2/SM3 tumors was 4.265 ± 3.233 (*P *<* *.0001). Among 51 pT1a‐EP/LPM tumors, 10 (19.6%) showed positive detection of FDG. For pT1a‐MM/pT1b‐SM1 and pT1b‐SM2/SM3 tumors, the detection rate was 52.9% (18/34) and 82.0% (50/61), respectively. The detection rate of pT1a‐EP/LPM was significantly lower than in the other two groups (*P *<* *.0001). Among 10 FDG‐PET‐positive lesions, only 1 had no apparent reason for PET positivity; however, 9 of 10 had a suitable reason for detectability by PET and inadequacy for ER. Negative detection of superficial esophageal squamous cell carcinoma by FDG‐PET is useful to determine the indication for ER when the tumor depth cannot be diagnosed even after performing magnifying endoscopy with narrow band imaging and endoscopic ultrasonography. When FDG uptake is recognized, a therapeutic modality other than ER should be considered.

## 1 INTRODUCTION

Esophageal cancer is one of the most aggressive cancers of the digestive tract.^1^ Historically, the most important therapy has been esophagectomy with extended lymphadenectomy.^2^, ^3^ However, recent developments in gastrointestinal endoscopy have enabled the detection of superficial esophageal cancer, and endoscopic resection (ER), especially endoscopic submucosal dissection (ESD), has become a standard therapeutic modality for mucosal esophageal cancer.^4^, ^5^, ^6^ In Japan, the indication for ER of esophageal cancer is a tumor located within the mucosal epithelium (T1a‐EP [equal to Tis in the TNM classification^7^]) or lamina propria mucosae (T1a‐LPM) because the incidence of lymph node metastasis is very low (≤5%).^8^ The recommended diagnostic modalities are endoscopic ultrasonography (EUS)^9^ and magnifying endoscopy with narrow band imaging (ME‐NBI).^10^ However, when these modalities are used, it is sometimes difficult to accurately determine the depth of tumor invasion of superficial esophageal cancer.

^18^F‐Fluorodeoxyglucose positron emission tomography (FDG‐PET) is a useful imaging modality that reflects the tumor volume and activity.^11^, ^12^ Because of its limited spatial resolution, FDG‐PET has not been used for superficial cancer but has instead mainly been used for advanced cancer.^13^ However, the lack of FDG accumulation might help to determine the indications for esophageal ESD.

In this study, we investigated the relationship between the depth of superficial esophageal cancer and the accumulation of FDG with the aim of determining the indications for ER.

## 2 MATERIALS AND METHODS

### 2.1 Patients

From 2009 to 2017, 444 patients with esophageal cancer underwent esophagectomy or ESD in Dokkyo Medical University Hospital, Tochigi, Japan. Among them, 195 patients had pathological superficial (mucosal or submucosal) cancer. Among these 195 patients with superficial cancer, 146 were examined by FDG‐PET before esophagectomy or ESD and were enrolled in this study. The characteristics of these 146 patients are summarized in Table 1. To stage the tumor in accordance with the TNM classification (7th edition) of the Union for International Cancer Control,^14^ all patients underwent esophagogastroduodenoscopy including ME‐NBI and EUS (with a 20‐MHz probe), computed tomography (CT) scanning, and FDG‐PET/CT scanning from the neck to the abdomen. All patients provided written informed consent for data access according to our institutional guidelines. This study was approved by the institutional review board.

**Table 1:** Patients’ characteristics

Factors	n
Age (mean ± SD)	67.1 ± 9.7
Sex	
Male	125
Female	21
Histology	
Squamous cell carcinoma	126
Basaloid carcinoma	5
Adenosquamous carcinoma	1
Adenocarcinoma	7
Barrett's adenocarcinoma	6
Carcinosarcoma	1
Location	
Upper	22
Middle	77
Lower	47
Depth of tumor invasion	
T1a‐EP (Tis)	24
T1a‐LPM	27
T1a‐MM	23
T1b‐SM1	11
T1b‐SM2	27
T1b‐SM3	34
Lymph node metastasis	
N0	127
1	16
2	3
3	0
Stage	
0	25
IA	103
IIB	15
IIIA	3
Excisional method	
ESD	50
Esophagectomy	96

ESD, endoscopic submucosal dissection; SD, standard deviation.

### 2.2 PET/CT protocol

The PET/CT was performed about 2 weeks before esophagectomy or ESD with an integrated scanner (Biograph 16 or Biograph LSO scanner; Siemens, Erlangen, Germany). All patients fasted for at least 6 hours. Before administration of FDG, a blood glucose level of <150 mg/dL was required. Whole‐body images were obtained approximately 60 minutes after intravenous administration of ^18^F‐FDG at a dose of 4.5 MBq/kg body weight (up to 450 MBq). Imaging was performed in six to eight bed positions based on the patient's height. Low‐dose CT was performed (nine effective mAs) to reduce radiation exposure.

### 2.3 Imaging assessment by PET/CT

The primary tumor was assessed by PET/CT, and the maximum standardized uptake value (SUV_max_) was measured. The SUV_max_ was measured by setting the region of interest with syngo.via software (Siemens Healthcare, Malvern, PA, USA). The SUV was defined as follows: SUV = radioactive concentration in tissue or lesion (MBq/g)/injected dose (MBq)/patient's body weight (g).

In this series, we defined the cutoff value as a SUV_max_ of 1.0 (the same as the background level).

### 2.4 Endoscopic assessment using ME‐NBI and EUS

The depth of tumor invasion of all lesions was estimated prior to treatment using a magnifying endoscope (GIF‐H260Z; Olympus, Tokyo, Japan) combined with NBI. According to the classification established by the Japan Esophageal Society, abnormal microvessels in cancerous lesions were categorized as type B. Type B vessels were subclassified into B1, B2, and B3, which were the diagnostic criteria for T1a‐EP or T1a‐LPM, T1a‐MM or T1b‐SM1, and T1b‐SM2 or deeper tumors, respectively.^9^ Additionally, EUS was performed using a 20‐MHz catheter probe (UM‐S20‐17S; Olympus) and the water filling method.

### 2.5 Statistical analysis

The chi‐square test and Fisher's exact test were used for statistical comparisons of nominal variables where appropriate. Continuous variables are expressed as mean ± standard deviation and were analyzed by one‐way analysis of variance. A post‐hoc test was performed by Bonferroni correction. The level of statistical significance was set at *P *<* *.05. All statistical analyses were carried out using R software (version 3.3.1).

## 3 RESULTS

### 3.1 Relationship between FDG uptake in primary tumor and depth of tumor invasion

**Figure 1:**
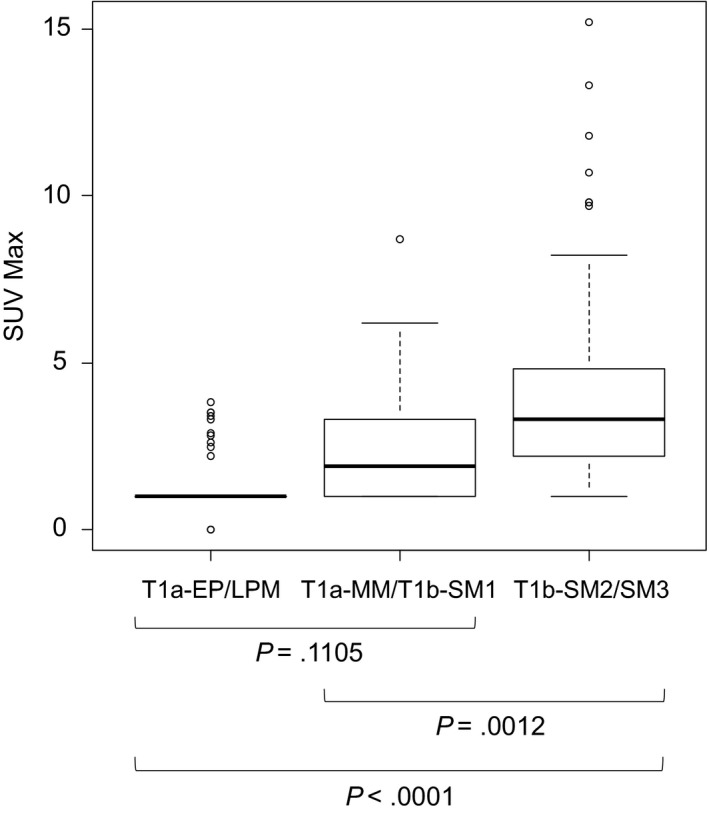
Relationship between SUV_max_ and depth of tumor invasion among superficial esophageal cancers. The mean SUV_max_ for pT1a‐EP/LPM tumors was 1.362, that for pT1a‐MM/pT1b‐SM1 tumors was 2.453, and that for pT1b‐SM2/SM3 tumors was 4.265

The mean SUV_max_ of the 146 pT1 tumors was 2.829. These pT1 tumors were divided to three groups: pT1a‐EP/LPM (n = 51), pT1a‐MM/pT1b‐SM1 (n = 34), and pT1b‐SM2/SM3 (n = 61) according to the differences in recommended therapy by the Japanese guideline.^8^ Thus, the mean SUV_max_ for pT1a‐EP/LPM was 1.362 ± 0.890, that for pT1a‐MM/pT1b‐SM1 was 2.453 ± 1.872, and that for pT1b‐SM2/SM3 was 4.265 ± 3.233. There was a significant positive correlation between the SUV_max_ and pathological tumor depth (*P *<* *.0001) (Figure 1).

### 3.2 Detection rate of FDG uptake according to depth of tumor invasion

**Figure 2:**
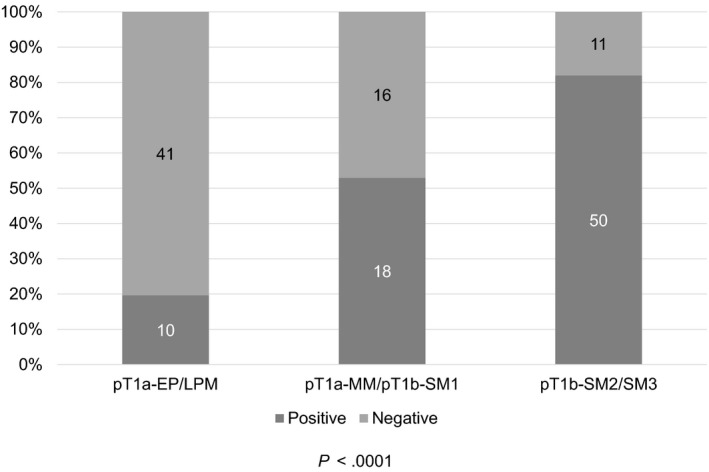
Detection rate of FDG according to depth of tumor invasion, a total of 19.6% (10/51) of pT1a‐EP/LPM tumors, 52.9% (18/34) of pT1a‐MM/pT1b‐SM1 tumors, and 82.0% (50/61) of pT1b‐SM2/SM3 tumors showed positive detection of FDG

Among the 146 superficial esophageal cancer lesions, 78 (53.4%) were detected by FDG‐PET. The analysis according to tumor depth showed positive detection of FDG in 10 (19.6%) of 51 pT1a‐EP/LPM tumors, 18 (52.9%) of 34 pT1a‐MM/pT1b‐SM1 tumors, and 50 (82.0%) of 61 pT1b‐SM2/SM3 tumors. The detection rate in pT1a‐EP/LPM tumors was significantly lower than that in the other two groups (*P *<* *.0001) (Figure 2).

### 3.3 Characteristics of pT1a‐EP/LPM with positive detection of FDG

**Figure 3:**
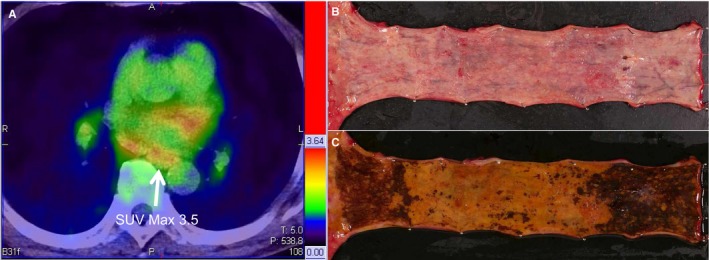
A case of superficial extensive spreading‐type SCC. A, FDG‐PET image. FDG uptake (SUV_max_ = 3.5) was detected in the thoracic esophagus. B, Resected specimen. Superficial flat‐type tumor spread in the thoracic esophagus. C, Iodine staining of resected specimen. An entire circumferential and 103‐mm‐long superficial tumor was evident by iodine staining. Pathological examination revealed that the depth of tumor invasion was T1a‐EP

The details of pT1a‐EP/LPM with positive detection of FDG are shown in Table 2. Ten lesions were diagnosed as PET positive. Four of them were superficial extensive spreading‐type squamous cell carcinoma (SCC) (Figure 3). Two were Barrett adenocarcinoma with inflamed mucosa due to gastroesophageal reflux (GER). One lesion was not Barrett adenocarcinoma but instead circumferential SCC with a 4‐cm length at the esophagogastric junction with inflamed mucosa due to GER. Two lesions had undergone a PET scan 2 days after esophageal biopsy in the endoscopic examination. Only one lesion had no apparent reason for PET positivity. That is, 9 of the 10 lesions had a suitable reason for why the lesion was detectable by PET.

**Table 2:** Details of pT1a‐EP/LPM lesions with FDG uptake

Case	SUV_max_	Age	Sex	Location	Tumor type	Histology	pT	ly	v	Tumor size (cm)	Feature	Method
1	2.2	59	M	Middle	0‐IIb	SCC	T1a‐LPM	0	0	10 × 5	Superficial extensive spreading‐type	Esophagectomy
2	2.47	59	M	Lower	0‐Is	Barrett	T1a‐LPM	1	0	3 × 1.7	Barrett adenocarcinoma with GERD	Esophagectomy
3	2.6	70	M	Upper	0‐IIb	SCC	T1a‐LPM	0	0	14.7 × 5	Superficial extensive spreading‐type	Esophagectomy
4	2.8	74	F	Lower	0‐IIb + IIc	SCC	T1a‐EP	0	0	3 × 4.3	Circumferential SCC on EGJ with GERD	Esophagectomy
5	2.87	74	M	Middle	0‐IIc + IIa	SCC	T1a‐LPM	0	0	3.2 × 1.5	Two days after esophageal biopsy	ESD
6	3.3	71	F	Middle	0‐IIa	SCC	T1a‐EP	0	0	10 × 5	Superficial extensive spreading‐type	Esophagectomy
7	3.4	71	M	Middle	0‐IIc	SCC	T1a‐LPM	0	0	1.4 × 0.7	Two days after esophageal biopsy	Esophagectomy
8	3.5	66	M	Middle	0‐IIb + IIc	SCC	T1a‐EP	0	0	12 × 4.5	Superficial extensive spreading‐type	Esophagectomy
9	3.5	71	M	Middle	0‐IIa	SCC	T1a‐LPM	0	0	2.5 × 1.6	—	ESD
10	3.8	44	M	Lower	0‐IIb	Barrett	T1a‐EP	0	0	2.7 × 5	Barrett adenocarcinoma with GERD	Esophagectomy

ly, lymphatic invasion; v, venous invasion; EGJ, esophagogastric junction; ESD, endoscopic submucosal dissection; FDG, fluorodeoxyglucose; GERD, gastroesophageal reflux disease; SCC, squamous cell carcinoma; SUV_max_, maximum standardized uptake value.

### 3.4 Comparison between PET and endoscopic diagnosis of pT1a‐EP/LPM SCC

**Figure 4:**
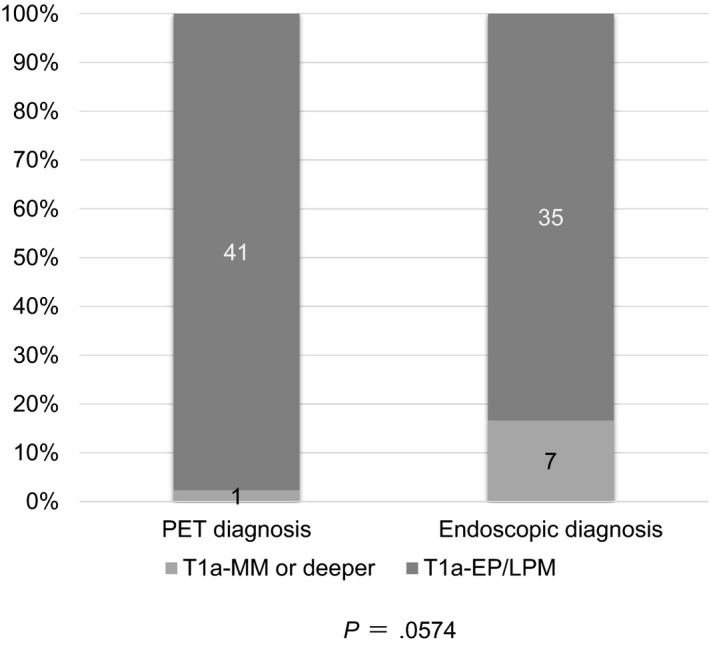
Comparison between PET and endoscopic diagnosis for pT1a‐EP/LPM SCC. In total, 41 of 42 lesions (97.6%) were diagnosed as T1a‐EP/LPM; in contrast, after endoscopic diagnosis with ME‐NBI and EUS, 35 of 42 lesions (83.3%) were diagnosed as T1a‐EP/LPM

From the above results, the efficacy of FDG‐PET diagnosis of pT1a‐EP/LPM SCC was examined. That is, 42 lesions excluding superficial extensive spreading‐type SCC, SCC with inflamed mucosa due to GER, SCC just after endoscopic biopsy, and Barrett adenocarcinoma were examined. The lesion without FDG uptake was considered to correspond to pT1a‐EP/LPM. According to this definition, 41 of 42 lesions (97.6%) were diagnosed as T1a‐EP/LPM. Endoscopic diagnosis with ME‐NBI and EUS was examined for comparison, and 35 of 42 lesions (83.3%) were diagnosed as T1a‐EP/LPM. PET diagnosis tended to be more accurate than endoscopic diagnosis although the difference was not statistically significant (*P *=* *.0574) (Figure 4).

## 4 DISCUSSION

The FDG‐PET is a superior modality for detecting malignant lesions in the digestive tract and other sites.^15^, ^16^, ^17^, ^18^, ^19^ FDG‐PET can be used for imaging of tumor activity by reflecting glucose metabolism; rarely, it can also be used to obtain a functional imaging diagnosis of malignancy. However, its spatial resolution is limited. Kato et al^20^ reported that the minimal diameter of detectable malignant lesions of the esophagus is 5 mm. Therefore, FDG‐PET has not been considered useful for detection of minimal or superficial cancer. However, tumors that cannot be detected by FDG‐PET may have less malignant potential. Accordingly, we assumed that negative FDG‐PET findings can be used to determine the indication for ER.

Esophageal cancer is the most aggressive malignant neoplasm among those in the digestive tract. The most important prognostic factor is lymph node metastasis.^21^ Among superficial esophageal cancers, the incidence of lymph node metastasis associated with T1a‐EP and T1a‐LPM tumors is very low (≤5%), that associated with tumors located within the muscularis mucosa (T1a‐MM) and tumors invading the upper third of the submucosal layer (T1b‐SM1) ranges from 10% to 20%, and that associated with tumors invading the middle and deeper third of the submucosal layer (T1b‐SM2, T1b‐SM3) may reach 50%.^22^ Therefore, T1a‐EP and T1a‐LPM tumors are absolute indications for ER. T1a‐MM and T1b‐SM1 tumors are relative indications for ER, and when such a depth is confirmed pathologically, additional therapy (esophagectomy or chemoradiotherapy) is considered. For T1b‐SM2 and T1b‐SM3 tumors, esophagectomy accompanied by lymphadenectomy is recommended as the standard therapy.

However, distinguishing T1a‐EP/LPM from T1a‐MM/T1b‐SM1 is sometimes difficult. ME‐NBI has recently been used to determine the depth of SCC invasion in Japan.^8^ ME‐NBI is a diagnostic modality that utilizes pattern recognition of abnormal microvessels. Although this modality has a high level of diagnostic accuracy, some cases are difficult. EUS has been utilized as another modality to confirm the depth of tumor invasion. High‐frequency EUS is used to detect the fine layer structure of superficial esophageal cancer.^23^ However, this modality is easily affected by esophageal contraction and therefore tends to have less reproducibility.

In the present study, 41 of 51 pT1a‐EP/LPM cases (80.4%) showed no FDG uptake and 10 cases showed slight FDG uptake. Among them, four were superficial extensive spreading‐type SCC. According to the Japan Esophageal Society, superficial extensive spreading‐type SCC is defined as a superficial type 0‐II tumor in which the maximal length of the tumor extends ≥5 cm longitudinally.^24^ FDG uptake partially reflects the tumor volume, and superficial extensive spreading‐type SCC has a certain tumor volume. Therefore, such tumors can probably be detected by FDG‐PET. Superficial extensive spreading‐type SCC is difficult to dissect endoscopically and reportedly has higher malignant potential than usual superficial SCC. Therefore, this tumor is thought to be a poor indication for ER. Two patients had Barrett adenocarcinoma with inflamed mucosa due to GER. These tumors were not SCC and had a clear reason for FDG uptake. One lesion was not Barrett adenocarcinoma but instead was a circumferential SCC with a 4‐cm length at the esophagogastric junction and mucosal inflammation due to GER. In addition to the presence of inflammation, a circumferential lesion at the esophagogastric junction is associated with a high risk for cicatricial stenosis after ER. Accordingly, this lesion may also be a poor indication for ER. The other two cases were just after esophageal biopsy in the endoscopic examination. One showed no apparent reason for FDG uptake. That is, only 1 of 42 usual pT1a‐EP/LPM SCCs (3.3%) had positive detection by FDG‐PET.

Thus, negative FDG‐PET findings for superficial SCC are thought to be helpful to determine the indication for ER. Conversely, Kita et al^25^ reported that if FDG uptake is recognized, a therapeutic modality other than ER should be considered.

In principle, our indication for ESD is clinical T1a‐EP and LPM. When required, T1a‐MM and T1b‐SM1 are included. Among 50 patients who underwent ESD, 39 had clinical T1a‐EP and LPM. Of these 39 patients, 31 (79.5%) had pathological T1a‐EP and LPM. If the FDG‐PET criteria are applied to these patients, 36 would have clinical T1a‐EP and LPM, and the diagnostic accuracy would become 31/36 (86.1%). Thus, the diagnostic accuracy must improve if we use FDG‐PET before ESD.

**Figure 5:**
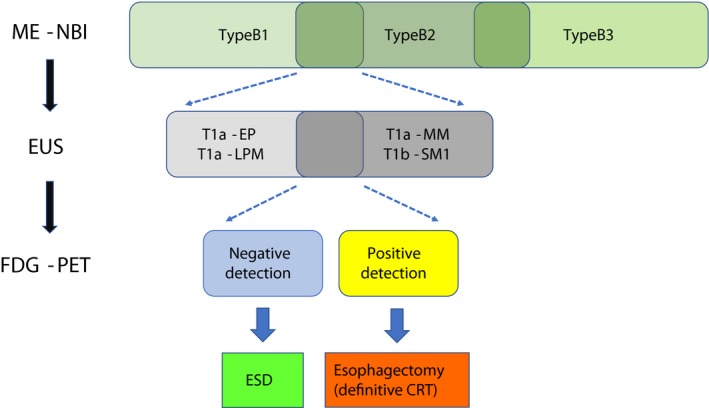
Proposed diagnostic flow for superficial SCC. First, endoscopic examination using ME‐NBI should be performed. When type B1 vessels must be differentiated from type B2 vessels, EUS should be performed as the second diagnostic modality. In patients with subtle superficial tumors that are difficult to diagnose as T1a‐EP/LPM after this two‐step examination, FDG‐PET should be considered. In cases of negative detection, ESD should be performed; in cases of positive detection, esophagectomy or definitive CRT should be considered

However, FDG‐PET has some limitations. For example, it is expensive to perform, and patients are exposed to minimal internal irradiation. Therefore, it does not seem advisable to perform FDG‐PET in all patients with superficial esophageal cancer in clinical practice. Actually, we believe that the best modality with which to diagnose T1a‐EP/LPM carcinoma is ME‐NBI and that the second best modality is EUS. Therefore, FDG‐PET should be considered a complementary diagnostic technique when the physician cannot determine the tumor depth even after performing ME‐NBI and EUS; FDG‐PET will benefit such patients with subtle borderline tumors. When treating patients with superficial esophageal cancer, the cost‐effectiveness of FDG‐PET will increase by limiting the technique to such patients. We propose a diagnostic flow for superficial esophageal SCC in Figure 5.

In conclusion, negative detection of superficial esophageal SCC by FDG‐PET is useful to determine the indication for ER. If the spatial resolution is improved, FDG‐PET will have more advantages in the diagnosis of esophageal cancer.

## CONFLICT OF INTEREST

All authors have no conflict of interest.
